# The Effect of Propofol on Mitochondrial Fission during Oxygen-Glucose Deprivation and Reperfusion Injury in Rat Hippocampal Neurons

**DOI:** 10.1371/journal.pone.0165052

**Published:** 2016-10-27

**Authors:** Haibin Wang, Shengfa Zheng, Maodong Liu, Changxin Jia, Shilei Wang, Xue Wang, Sha Xue, Yunliang Guo

**Affiliations:** 1 Department of Anesthesiology, the Affiliated Hospital of Qingdao University, Huangdao, Qingdao, Shandong Province, China; 2 Institute of Cerebrovascular Diseases, Affiliated Hospital of Qingdao University, Huangdao, Qingdao, Shandong Province, China; 3 Department of Anesthesiology, People's Hospital of Rizhao, Rizhao, Shandong Province, China; Indian Institute of Integrative Medicine CSIR, INDIA

## Abstract

The neuroprotective role of propofol in transient global and focal cerebral ischemia reperfusion (I/R) animal model has recently been highlighted. However, no studies have conducted to explore the relationship between mitochondrial fission/fusion and I/R injury under the intervention of propofol. Moreover, neuroprotective mechanism of propofol is yet unclear. Culturing primary hippocampal cells were subjected to oxygen-glucose deprivation and re-oxygenation (OGD/R) model, as a model of cerebral I/R in vitro. Methods CCK-8 assay was used to test the effect of propofol on cell viability. We examined the effect of propofol on mitochondrial ultrastructure and mitochondrial fission evoked by OGD/R with transmission electron microscopy and immunofluorescence assay. To investigate possible neuroprotective mechanisms, the authors then examined whether propofol could inhibit calcium-overload, calcineurin (CaN) activation and the phosphorylation of dynamin-related protein 1 (Drp1) during the period of OGD/R, as well as the combination of Drp1-ser 637 and fission 1 (Fis1) protein by immunofluorescence assay, ELISA and double-labeling immunofluorescence analysis. Finally, the expression of Drp1-ser 637 and Fis1, apoptosis inducing factor (AIF) and cytochrome C (Cyt C) were detected by western blot. When added in culture media during OGD period, propofol (0.1μM-50μM) could alleviate neurons injury and protect mitochondrial ultrastructure, meanwhile inhibit mitochondrial fission. Furthermore, the concentration of intracellular free Ca^2+^, CaN activition and the phosphorylation of Drp1-ser637 were suppressed, as well as the translocation and combination of Drp1-ser 637 and Fis1. The authors also found that the expression of Cyt C, AIF, Drp1-ser637 and Fis1 were down-regulated. Notably, high dose of propofol (100μM-200μM) were confirmed to decrease the survival of neurons based on results of cell viability. Propofol could inhibit mitochondrial fission and mitochondrial apoptotic pathway evoked by OGD/R in rat hippocampal neurons, which may be via depressing calcium-overload.

## Introduction

Ischemic brain injury, a serious occurring complication of shock, cardiac arrest, cardiopulmonary bypass or accidents during operation and anesthesia, is a main cause of death and disability in adults[1]. The key treatment of ischemic injury is to reestablish blood supply for ischemic region in its narrow therapeutic time windows. But the doctor would not accept these therapies because of the further injury followed by reperfusion[2]. Recent studies have suggested that anesthetic drugs have neuroprotective effects on cerebral I/R injury via reducing cerebral blood flow(CBF) and cerebral metabolic rate of oxygen(CMRO)[3–4], and extensive experimental confirmed it. For instance, it has been reported that dexmedetomidine ameliorated hepatic I/R injury by acting on lipid peroxidation and cellular membrane alterations and kidney I/R injury via inhibiting JAK2/STAT3 signaling pathway partially[5–6], and preconditioning with isoflurane reduced ischemia-induced brain injury may be involved mitochondrial adenosine 5'-triphosphate-sensitive potassium channels in rats[7]. Moreover, it has been confirmed that propofol reduced ischemic brain injury via gultamatergic signaling pathway in rats[8].

Propofol (2, 6-diisopropylphenol), an intravenous sedative–hypnotic agent, is widely used in anesthesia induction, maintenance and intensive care. It has been found to have neuroprotective effect on cerebral I/R animal models[9–10]. Compared to other anaesthetic, it could reduce both CBF and CMRO, and could not cause the risk of increasing intracranial pressure(ICP). The neuroprotective mechanisms of propofol have been suggested to including its antioxidant properties toward radical[11], inhibition of calcium overload[12], activation of γ-aminobutyric acid receptors[13], suppression of N-methyl-D-aspartate receptors[14–15]and its anti-apoptosis programs[16].

It is important to keep a balance between mitochondrial fission and fusion to protect its functions, which are toughly related to apoptosis during cerebral I/R injury[17–19]. Our previous study had reported that mitochondrial fission were increased during apoptosis and mdivi-1(a selective inhibitor of Drp1), could protect neurons from cerebral I/R injury via inhibiting mitochondrial fission in rats and rat hippocampal neurons[20]. Propofol could reduce OGD-induced neuron mitochondrial swelling by preventing the mitochondrial membrane permeability increasing[21]. But no further research is conducted to explore the effect of propofol on mitochondrial fission/fusion during cerebral I/R injury.

In this study, we explored the effects of propofol on mitochondrial fission in rat primary hippocampal cells subjected to OGD/R and investigated its neuroprotective mechanisms. We aimed to examine whether propofol could inhibit calcium overload, the phosphorylation of Drp1-ser637 and its translocation to outer mitochondrial membrane. And whether it could play a role in controlling mitochondrial apoptotic pathway by inhibiting the expression of mitochondrial apoptosis factors to counteract the ischemia injury induced by OGD/R in CA1 pyramidal cells.

## Materials and Methods

### Primary Hippocampal Neuronal Culture and Experimental Groups

This study was approved by the institution of animal care and use committee at the Ethics Committee of Qingdao University Medical College. 626 Sprague-Dawley rats, born within 24h were supplied by the Experimental Animal Center of Qingdao Drug Inspection Institute. The primary hippocampal neurons, which have been extensively used in our laboratory, were cultured as previously described by Banker GA[22] with some modifications. After rat pups were surface sterilized with 75% alcohol and then sacrificed by cervical dislocation, the authors removed hippocampi from the entire brain of rats on ice, and divested vessels carefully, then mechanically fragmented and trypsinized the tissues for 20–30 min at 37°C. Finally, isolated cells were plated on poly-lysine-coated 25-mm Petri dishes at a density of 700,000 cells.

The cells were cultured with DMEM/F12 solution mixed with 20% fetal bovine serum at 37°C in a humidified incubator with 95% (vol·vol^-1^) O_2_ and 5% (vol·vol^-1^) CO_2_. 24 hours after plating, half of culture media was replaced with the same volume of Neurobasal-A medium supplemented with B27 (2%), 1 mol·L^-1^ glutamine (1%) and 0.1 mol·L^-1^ sodium pyruvate (1%). Half of the media was replaced by the same fresh solution every other day. We observed the growth of cells under inverted phase contrast microscope everyday. After 8day cultivation, the primary hippocampal neurons were identified by NSE staining under fluorescent microscopy (Leica, DMI 4000 B, Japan).

The cultured neurons were randomly divided into 8 groups:

1. Control group: cultured normally without any treatment;
2. Vehicle group: dimethylsulfoxide (DMSO, 0.1% final concentration) was added to culture media during OGD/R period of I/R group;
3. I/R group: after 8-day cultivation, neurons were subjected to OGD for 6h followed by re-oxygenation for 20h but given no drugs;
4. I/R+1μM propofol (P1 group): the culture medium mixed with propofol (1μM) during OGD/R period;
5. I/R+10μM propofol (P2 group): the culture medium mixed with propofol (10μM) during OGD/R period;
6. I/R+50μM propofol (P3 group): the culture medium mixed with propofol (50μM) during OGD/R period;
7. I/R+100μM propofol (P4 group): the culture medium mixed with propofol (100μM) during OGD/R period;
8. I/R+200μM propofol (P5 group): the culture medium mixed with propofol (200μM) during OGD/R period.

### Oxygen-Glucose Deprivation (OGD) Model and Drug Administration

Propofol (purity>99%, Sigma), dissolved in DMSO as a stock solution, was added during OGD period in serum-free culture media (0.1% final concentration). Primary hippocampal neurons were rinsed twice with phosphate-buffered saline to flush off any previous culture medium on the eighth day which was the most sensitive stage for hypoxia. In order to build the model of OGD, neurons were exposed to glucose-free EBSS for 6 hours at 37°C in a humidified incubator with 2% (vol·vol^-1^) O_2_, 5% (vol·vol^-1^) CO_2_, and 93% (vol·vol^-1^) N_2_, then samples were changed back to the prior incubator with Neurobasal-A media. The treatment of vehicle group was the same as the normal group except for adding DMSO (0.1% final concentration). During the period of OGD/R, propofol were added to media with different concentration gradients (1μM, 10μM, 50μM, 100μM, 200μM), which respectively formed the group of I/R+P1, I/R+P2, I/R+P3, I/R+P4, I/R+P5.

### Cell Viability Assay

Cell viability was assessed by cell counting kit. Cells were cultured in 96-well plates at a density of 1×10^4^ per well. Each well was added with 100μl culture medium mixed with 10μl of 7Sea-Cell Counting kit and incubated for 2h with 95% (vol·vol^-1^) O_2_ and 5% (vol·vol^-1^) CO_2_. Absorbance was measured using a microplate reader at 450nm. Cell viability rate equals to (OD treatment/OD control) ×100%. Each experiment was repeated at least 3 times.

### Calcium Analysis

The concentration of intracellular free Ca^2+^ was measured by fluorescent dye Fluo-3 AM, which can enter the cells with the help of Pluronic F127, and decomposed into Fluo-3 by intracellular esterase. What’s more, the specific binding of Fluo-3 and Ca^2+^ formed chelates, which will produce a bright fluorescent signal with an excitation wavelength of 488 nm. Dissolved in DMSO, Pluronic F127 was added to Fluo-3 AM working solution, which diluted with Hanks’ solution to form working solution. The final concentration of Fluo3-AM and Pluronic F127 was 5μM and 0.05% (W·V^-1^) respectively. The samples were incubated with working solution away from light in 37°C for 30 min. The samples were then washed with Hanks’ solution for three times. After incubating in 37°C for 30 min again, the samples were determined by laser scanning confocal microscope (LSCM) under the excitation 488nm and emission 525nm to detect fluorescence intensity.

### Measurements of CaN Activition

CaN activition were determined by the luciferin–luciferase method, using a CaN detection kit (Nanjing Jiancheng Bioengineering Institute, Nanjing, China). Briefly, after treating cells with RIPA lysis buffer, we collected cell lysis solution by centrifugation at 12000r·min^-1^ for 10min at 4°C. The supernatant was used to detect the enzymatic activities and protein content. According to the manufacturer’s instructions, protein concentration was determined using the BCA method. Computation method of CaN was given in the direction.

### Transmission Electron Microscopy

Mitochondrial ultrastructure of neurons was observed by a JEM-1200EX transmission electron microscope (JEOL, Tokyo, Japan). Neurons collected by centrifugation were fixed with 1% (w·v^-1^) solution of osmium tetroxide. After embedded in Epon812-Araldite, the samples were cut into ultrathin slices (50nm thick) for observation.

### Mitochondria

Mitochondria were labelled by MitoTracker® Deep Red FM (Shanghai Yisheng Biotechnology Company, Shang Hai, China). The samples were incubated with working solution away from light in 37°C for 30 min. Then the samples were determined by LSCM with the excitation 644 nm and emission 665 nm to examine fluorescence intensity.

### Double-Labeling Immunofluorescence Analysis of Drp1-ser 637 and Fis1

To demonstrate the interaction between Drp1 and Fis1, we implemented double-labeling immuno-fluorescence analysis using primary antibodies anti-Drp1 antibody (rabbit polyclonal, Merck Millipore) and Fis1 (rat monoclonal, Merck Millipore) with the appropriate secondary antibodies goat anti-rabbit IgG/RBITC and goat anti-mouse IgG/FITC (Bioss, China). Samples were incubated for 30min at room temperature with a blocking buffer (5% bull serum albumin dissolved in TBST) and incubated overnight with the Drp1-ser637 antibody (1:200) and Fis1 antibody (1:200). Then they were washed with PBS buffer five times at 5min intervals and incubated for 1h with secondary antibody, followed by five additional washes at 5min intervals. Finally, the samples were determined at LSCM and analyzed by ImageJ.

### Western Blot

Western blotting, used to evaluate protein, was performed as described previously[23]. In brief, we extracted protein from cells and then measured protein concentrations using BCA kit. Protein samples were electrophoresed through 8% and 15% SDS-polyacrylamide gel and transferred onto polyvinylidene fluoride membranes. The membranes were then blocked with PBST which contains 5% non-fat milk for 2h on shaker. After that, membranes were incubated with the appropriate primary antibody over night at 4°C. The next day, membranes were washed in TBST for 3 times and incubated with HRP-conjugated secondary antibody for 1.5 hours. Finally, we washed the membranes again the same as last time and used the ECL chemiluminescence system (VILBER Fusion FX5 Spectra, France) to quantify the membranes by Electro-Chemi-Luminescence for enhancing the signals. Images were quantified by Quantity One Software (Bio-Rad, USA). GAPDH was served as the internal standard.

### Statistical Analysis

SPSS 18.0 was used to execute statistical analysis. Data were expressed as means±SD, and a difference was considered statistically significant when p<0.05. ANOVA was used to compare differences among groups.

## Results

### Effect of Propofol on Survival of Hypoxia-Reperfusionated Neurons

**Fig 1:**
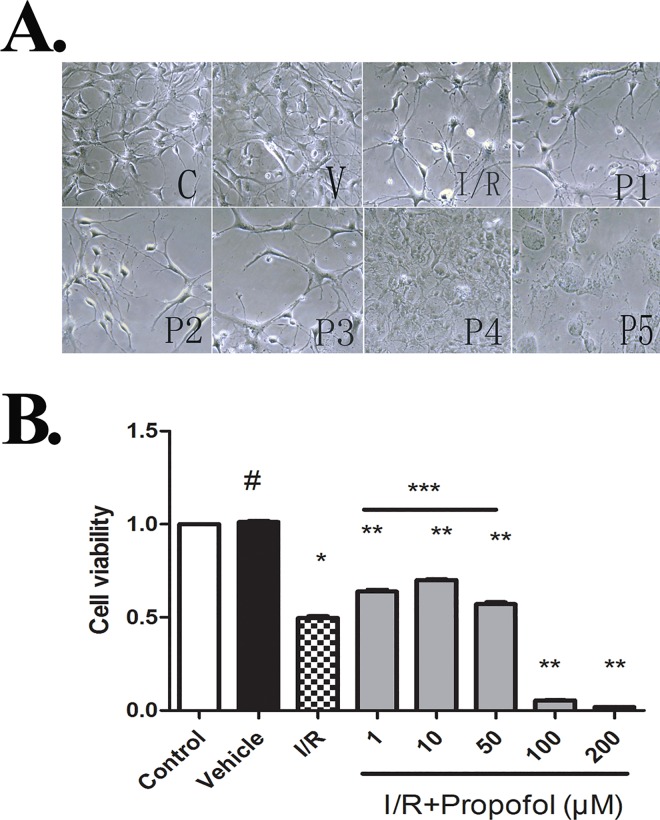
**(A) Neurons examined by phase contrast microscopy.** Photomicrographs displayed that OGD and reperfuson elicited obvious changes of neurons with disintegration of cell body and dendrites compared to control group. Propofol(1μM-50μM) added during OGD markedly alleviate I/R damages, While Propofol(100μM-200μM) showed a more severe injury compared to I/R group. **(B) Cell viability was assessed by the cell counting kit.** Neurons were plated at a density of 1×104 per well in triplicate wells of 96-well plats. Values are represented as the mean ± SD. The results showed no differences in cell viability that were observed between the control and vehicle groups (P^#^>0.05). In the groups subjected to OGD and reperfusion injury, cell viability was significantly lower than in the control and vehicle group (P*<0.01, versus control and vehicle group). In propofol treated groups, cell viability was apparently higher than that in I/R group(P**<0.05), and among propofol treated groups cell viability in 10 μM propofol treated group was higher than others(p***<0.05, versus P1+I/R group, P3+I/R group).

Morphological changes of neurons subjected OGD and reperfusion were observed by the phase contrast microscopy. Compared to normal cultures, OGD and reperfusion injury decreased the number of neurons. Propofol was added during OGD and 0.1μM-50μM rather than 100μM-200μM of propofol exhibited a neuroprotective effect against ischemic reperfusion injury (Fig 1A).

**Fig 2:**
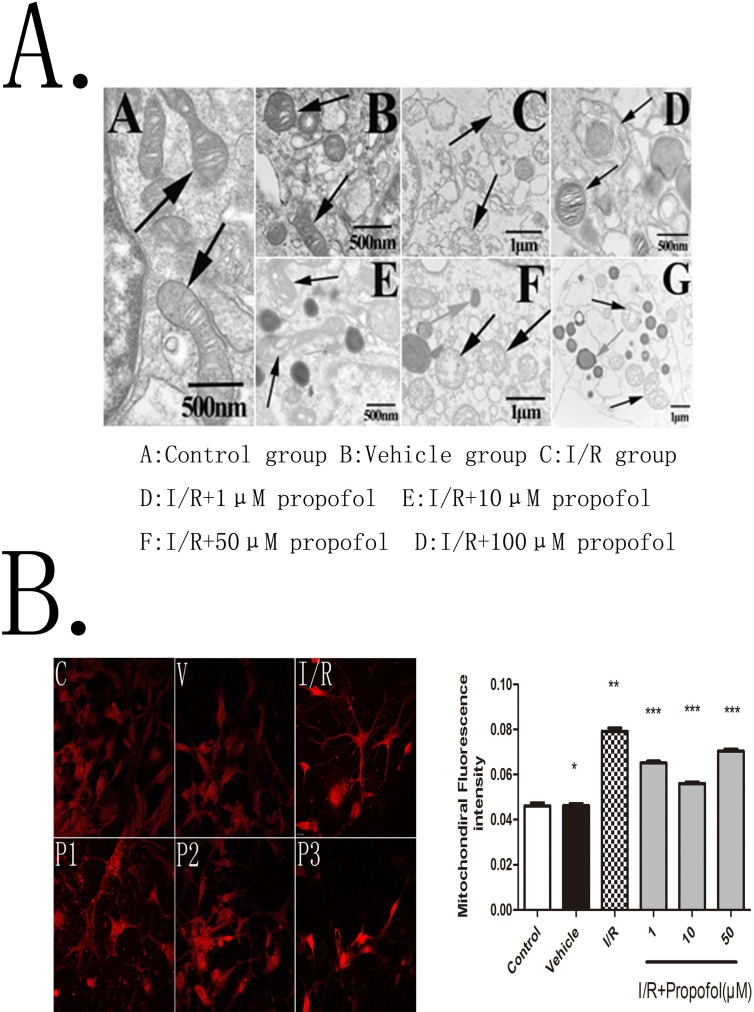
(**A) Electron microscopic evaluation of the mitochondrial ultrastructure.** The primary hippocampal neurons, cultured to 8th days, subjected(I/R, P1-P4) or not(C and V) to an oxygen–glucose deprivation(OGD) and treated or not(C, V, I/R) with propofol(1μM, 10μM, 50μM, 100μM) (P1-P4), and then observed by a transmission electron microscope. The pictures showed that the mitochondrial ultrastructure of control group and vehicle group were normally including complete andclear structure of mitochondrial cristae but the mitochondrial of others(I/R, P1-P4) which subjected to OGD, were all becomed swelling, vacuolization and rupture with cristae disruption. The ultrastructure damage of the mitochondria were relatively slight for the groups(P1-P3) treated with propofol, but the group of P4. **(B) Mitochondria** was dyed by MitoTracker® Deep Red FM and then determined at LSCM by the excitation 644 nm and emission 665 nm to examine fluorescence intensity. Values are represented as the mean ± SD. The mean flourscence indensity of mitochondria was markedly higher in I/R group. While the mean flourscence indensity was lower in I/R+propofol groups than in I/R group, and in I/R+propofol (10 μM) group had the minimum fluorescence intensity among I/R+propofol groups. P^#^>0.05, vs. control group; p*<0.01, vs. control group; p**<0.05, vs. I/R group; p***<0.05, vs. I/R+P1 and I/R+P2 groups.

As is shown in Fig 1B, neuron viability of the I/R group was lower than that in the control group and vehicle group. We can ignore the effects of DMSO on cells as there is no difference in cell viability between the control and vehicle group. Propofol (1μM-50μM) treated groups promoted cell survival obviously than I/R group, and 10μM propofol provided the best protection against I/R injury. However, the cell viability in groups treated with high dose of propofol (100μM and 200μM) was lower than that in I/R group. For the result of cell viability was in coincidence with photomicrographs (Fig 1A) and the changes of mitochondrial ultrastructure (Fig 2A), we abnegated the group of P4 (100μM) and P5 (200μM) in the followed experiments.

### Effect of Propofol on Mitochondrial Ultrastructure and Mitochondrial Fission

As is shown in Fig 2A, the ultrastructure of mitochondria changes in primary cultured hippocampal neurons observed by a JEM-1200EX transmission electron microscope (Jeol, Tokyo, Japan) at 20h after OGD. The normal mitochondria, as indicated by black arrow, which were intact with clear cristae, were caught in the control group. When experienced I/R injury, the pyramidal neurons performed typical signs of apoptosis, especially for the change of motochondria as shown in Fig 2A. As expected, the damage of neurons was obviously slight in the propofol treated groups (P1-P3). Among these concentrations, 10μM propofol has the best neuroprotective activity. And increasing the concentration of propofol to 100μM will be counterproductive to damage aggravation. These results revealed that propofol may performed neuroprotective effect by maintaining the stability of mitochondrial morphology during OGD and reperfusion injury. In addition, we also found that lipid droplets were appeared in propofol treated groups.

Our previous works had shown that mitochondrial fission would increase on model of cerebral I/R injury in vivo and in vitro^22, 38–39^. To determine whether the number of mitochondria was affected by propofol, the neurons was dyed by MitoTracker® Deep Red FM and then determined at LSCM by the excitation 644nm and emission 665nm to examine mitochondrial fluorescence intensity. Images (Fig 2B) showed that propofol partly reduced the number of mitochondria compared with I/R group. With the results above, it suggested that propofol could inhibit mitochondrial division and protect neurons from cerebral cerebral ischemia-reperfusion injury, and 10μM propofol provided the most effective neuroprotection (Fig 2B).

### Effect of Propofol on Intracellular Ca^2+^ Concentration and CaN Enzymatic Activities

**Fig 3:**
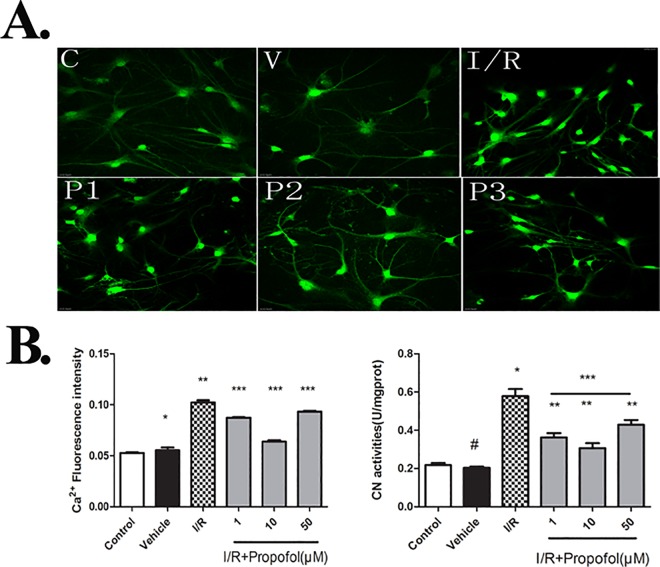
(A) **Ca**^**2+**^ fluointersity. **Ca**^**2+**^ was dyed by the explorer Fluo-3, AM and Pluronic F-127, then its concentration was determined using a Laser Scanning Confocal Microscope. Values are represented as the mean ± SD. The concentration of cytoplasmic free calcium was markedly higher in I/R group. Inversely, the concentration of cytoplasmic free calcium was lower in I/R+propofol(1μM-50μM) groups than in the I/R group. In addition, the concentration of cytoplasmic free calcium was lower in I/R+propofol (10 μM) group than in I/R+propofol(1μM and 50μM) groups. P^#^>0.05, vs. control group; p*<0.01, vs. control group; p**<0.05, vs. I/R group; p***<0.05, vs. I/R+P1 and I/R+P2 groups. **(B) Calcineurin enzyme activity.** It was determined by the luciferin–luciferase method, using the CN detection kit supplied by Nanjing Jiancheng Bioengineering Institute. Values are represented as the mean ± SD. The results showed that it is no differences between the control and vehicle groups in enzyme activity(P^#^>0.05). CN activity in the groups subjected to OGD and reperfusion injury were significantly higher than that in the control and vehicle group (P*<0.01, versus control and vehicle group). Propofol treatment significantly decreased CN activity(P**<0.01, versus I/R group), and CN activity in the 10 μM propofol was lower than that in the treatment with propofol(0.1μM, 50 μM)(p***<0.05, versus P1+I/R group, P3+I/R group).

To determine whether the cytoplasmic free calcium concentration is affected by propofol, the neurons were dyed using a fluorescent dye Fluo-3 AM, which can enter the cells with the help of Pluronic F127. The samples were determined using a Laser Scanning Confocal Microscope. As is shown in Fig 3A, calcium concentration of I/R group was significantly higher than that in control group and calcium concentrations in I/R+propofol groups were lower than that in I/R group, suggesting that propofol could prevent cytoplasmic calcium overload and thus reduce the severity of ischemic brain damage. In addition, 10μM propofol offered the most effective protection.

CaN activity is coupled to free calcium levels[24]. To review the relationship among CaN and calcium levels, enzyme activity was determined by the luciferin–luciferase method, using the CaN detection kit. There was no difference in enzyme activities between the control and vehicle groups (P>0.05). In I/R group, it was higher than that in the control group (P<0.01). Propofol treatment significantly decrease CaN activity (P<0.01), and it is consistent with the result of free calcium levels, 10 μM propofol treated group showed lower CaN activity than the other propofol groups, providing the best protection (Fig 3B).

### Effect of Propofol on the Expression and Colocalization of Fis1 and Drp1-ser637

**Fig 4:**
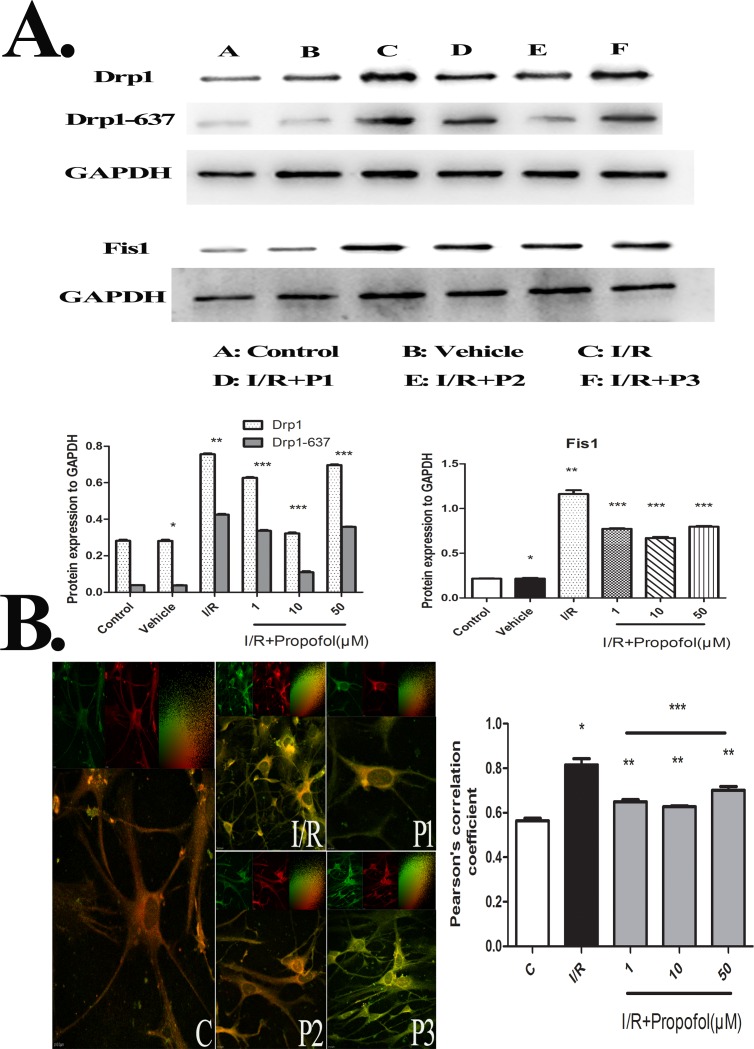
**(A) mitochondrial fission proteins**, Drp1 and Fis1 expression was detected by Western blot analysis after I/R injury. Values are expressed as the relative density and are represented as the mean ± SD (n = 3 per group). There were no differences in the levels of detected proteins between the control and vehicle group. In the I/R group, the levels of Drp1 and Fis1 increased significantly. However, pretreatment with propofol decreased the expression of Drp1 and Fis1. In addition, 10μM can minimize the expression of Drp1 and Fis1. P*>0.05, vs. control group; p*<0.01, vs. control group; p**<0.05, vs. I/R group; p***<0.05, vs. I/R+P1 and I/R+P2 groups. (B) **immunofluorescence of Fis1 (green) and Drp1-ser637(red) proteins,** showing the changes of their colocalization pattern following OGD and reperfusion with propofol administration (P1-P3) or not(I/R). Colocalization of Fis1 and Drp1-ser637 is revealed by the overlap of signals resulting in yellow staining. Embedded scatter gram in the upper left corner of the image estimates the amount of each detected antigen based on colocalization of Drp1-ser637(red, y-axis) and Fis1(green, x-axis). Colocalized pixels of yellow color are located along the diagonal of scatter gram. The pearson’s correlation coefficient, which describes the correlation of the intensity distribution between channels. It is from -1.0 to 1.0 and 0 indicates no significant correlation and -1.0 indicates complete negative correlation. It showed that the result of I/R group is apparently higher than control group. In propofol treated groups(P1-P3), it was lower than in I/R group, and it was no different between the group P1 and P2, but lower than P3 group. P*<0.01, vs. Control group; p**<0.05, vs. I/R group; p***<0.05, vs. P3 group.

To explore the neuroprotective molecular mechanisms of propofol via inhibiting I/R-induced mitochondrial fission, we examined the expression of mitochondrial fission proteins (Fig 4A). Drp1 and Fis1 are central factors to mitochondria fission. Compared to the control group, I/R injury significantly increased the expression of Drp1 and Fis1. However, when I/R group was pretreated with propofol (0.1μM-50μM), expression of these two proteins decreased significantly compared to I/R group. Among these concentrations of propofol, 10μM showed the most effective neuroprotection effect. It revealed that propofol might be through decreasing the expression of Drp1 and Fis1 to inhibiting mitochondial fission during I/R injury.

As is shown in Fig 4B, Confocal immunofluorescence microscopy of neurons stained with anti-Drp1-ser637 (red fluorescence) and anti-Fis1 (green fluorescence) antibodies. The pearson’s correlation coefficient which describes the correlation of the intensity distribution between channels, were exhibited on image B. It is from -1.0to 1.0 and 0 indicates no significant correlation and -1.0 indicates complete negative correlation. As Image (A) demonstrated that OGD and reperfusion injury caused a further increase of the combination of Drp1 and Fis1, while propofol could inhibit this combination of Drp1 and Fis1, especially for 1μM and 10μM propofol.

### Effect of Propofol on the Expression of Apoptosis Protein

**Fig 5:**
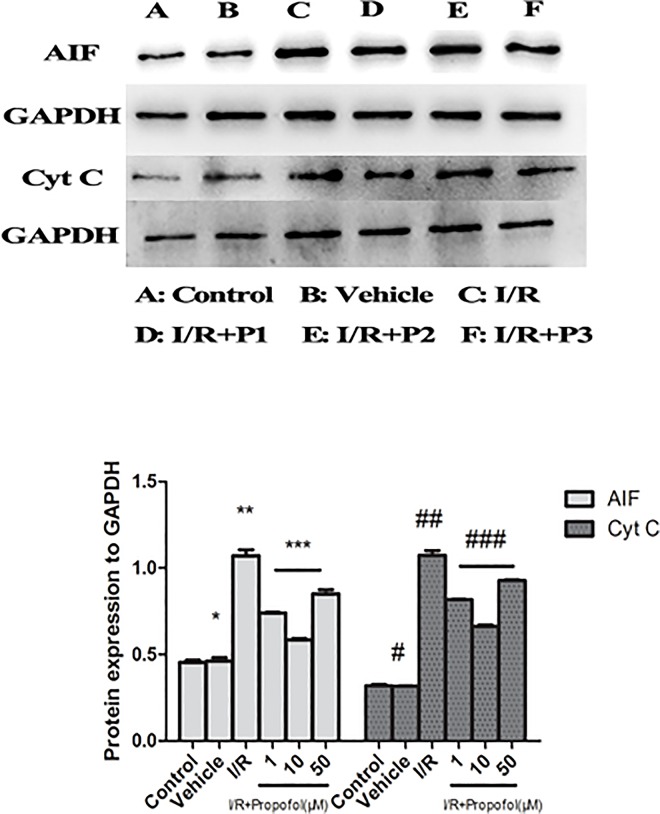
Apoptosis-related proteins, AIF and Cyt C expression was detected by Western blot analysis after I/R injury. Values are expressed as the relative density and are represented as the mean ± SD (n = 3 per group). There were no differences in the levels of detected proteins between the control and vehicle group. In the I/R group, the levels of AIF and Cyt C increased significantly. However, pretreatment with propofol decreased the expression of AIF and Cyt C. In addition, 10μM can minimize the expression of AIF and Cyt C. P*>0.05, vs. control group; p*<0.01, vs. control group; p**<0.05, vs. I/R group; p***<0.05, vs. I/R+P1 and I/R+P2 groups.

As is shown in Fig 5, we found that the expression of Cyt C and AIF corresponded to the results of Drp1 and Fis1. Propofol (0.1μM-50μM) decreased the expression of Cyt C and Fis1, and 10 μM is the optimal concentration to against cerebral I/R injury. We could presume that propofol could decrease the expression of Drp1 and Fis1 to inhibit mitochondrial fission, meanwhile, reducing Cyt C and Fis1 expression to suppress apoptosis in the process of I/R injury.

## Discussion

Recently, in vitro studies on neuroprotective mechanisms of propofol have paid attentions to morphology and structure of mitochondria on I/R model. Some relevant experiments[21,25–28]have preliminarily revealed that propofol could inhibit apoptosis by maintaining the stability of mitochondrial morphology and reducing the release of mitochondrial apoptosis related factors to cytoplasm. But there was no research designed to explore whether propofol could protect neurons from I/R injury through acting on the dynamic balance between mitochondrial fission and fusion, which had been proven to play a vital role on apoptosis during I/R injury[29].

In our study, we mimic brain I/R injury using OGD/R on primary cultured hippocampal neurons. The results showed that the injury to neurons after OGD/R involved mitochondrial swelling, rupture, and fragmentation (Fig 2). Propofol (1μM-50μM) can effectively reduce OGD/R-induced neuronal death 20h after the injury for partially reversing calcium-overload, decreasing mitochondrial swelling and fission during I/R injury (Figs 1–3). We also revealed that the probable mechanism may be that propofol inhibited mitochondrial fission by the phosphorylation of Drp1-Ser637 and its translocation to mitochondrion, which caused by CaN, activated by intracellular Ca^2+^ (Figs 2–4). Drp1 could not activate the mitochondrial division in the cytoplasm, only that Drp1 translocated to mitochondria and combined with Fis1. In contrast, once the translocation of Drp1-Ser637 to mitochondria were inhibited, the combination of Drp1 and Fis1 will be restricted[19]. What’s more, propofol (1μM-50μM) decreased the expression of Drp1 and Fis1 during OGD/R injury (Fig 4). Meanwhile, we also noticed that the expression of Cyt C, which was related to caspase-dependent programmed cell death (PCD), and AIF, which was related to caspase-independent PCD, were also suppressed by propofol (1μM-50μM) during OGD/R injury. These findings demonstrated that inhibiting mitochondrial fission and mitochondrial apoptotic pathway may be associated with neuroprotective effect of propofol during OGD/R injury.

The administration of propofol dose was based on the results of cell viability (Fig 1). The results demonstrated that 1μM-50μM propofol could offer protection effect against I/R injury in primary hippocampal cells, of which 10μM was the optimal dose. However, propofol concentrations above 100μM showed opposite impacts on neurons. Current studies on propofol concentrations of neuroprotection have yielded controversial results. A previous study had reported that propofol had sufficient neuroprotection effect on hypoxic hippocampal slice in both 5μM and 10μM propofol treatment groups[30], while further study showed that the concentrations of propofol 10–100μM considered to be higher than others, could attenuate CA1 injury in hippocampal slices exposed to OGD in vitro[21]. In addition, propofol provided dose-dependent protection against the OGD-induced cerebral injury[31]. Adembri C’s study[21] showed that propofol at clinically relevant concentrations had neuroprotective effects on models of cerebral ischemia in vitro and in vivo. In contrast, a study had indicated that 10μM and 100μM propofol had no neuroprotective on CA1 and CA3 hippocampal slices exposed to OGD[32]. We inferred that the disputing may result from diverse measurements and different theoretic models. Actually, the plasma protein binding ratio of propofol is so high that free concentration in plasma is quite low (0.22–1.8μM)[33]. In this study, at clinically relevant concentrations, 1μM propofol could offer neuroprotective effect on cerebral I/R injury, but the optimal concentration (10μM) was beyond clinically relevant concentrations. What’s more, a higher dosage of propofol had deleterious effects on mitochondria for impairments of oxidative phosphorylation[34].

Apoptosis is a major event leading to cerebral I/R injury and mitochondria playing a key role in I/R-induced apoptosis through integrating death signals and controlling the release of apoptogenic factors (such as Cyt C and AIF) from mitochondrial matrix to cytoplasm because of mitochondrial outer membrane becomes permeable[35–37]. Cyt C, a component of the electron transport chain, release from mitochondria to cytoplasm, which is crucial for activation of the mitochondrial apoptotic pathway to format the apoptosome composed by Cyt C, apaf-1, procaspase-9, and dATP[38]. AIF is a mitochondrial flavoprotein that will be released and induced caspase-independent PCD in response to death stimuli[39–40]. When AIF translocates to nuclei, it causes chromatin condensation, mitochondrial membrane depolarization and phosphatidylserine exposure on the cell surface[41]. In addition, previous studies had shown that the mitochondrial calcium uniporter (MCU) is a gateway to transport cytosolic Ca^2+^ into mitochondrial matrix[42–43], and our previous work also showed that MCU could modulate mitochondrial Ca^2+^ uptake during cerebral I/R injury[44]. Mitochondrial Ca^2+^ overload can induce mitochondrial membrane potential depolarization and the opening of mitochondrial permeability transition pore (MPTP), which results in the release of proapoptotic factors[45–49]. Moreover, we found that propofol (1μM-50μM) could decrease the expression of Cyt C and AIF. Therefore, we presumed that propofol could decrease the release of Cyt C and AIF by suppressing cytosolic calcium overload-induced mitochondrial membrane potential depolarization and inhibiting the expression of Cyt C and AIF.

For the first time, Frank[50] found that mitochondrial fission exerts important action in apoptosis upon inhibition of Drp1 to block cell death. Mitochondrial cristae will be remodeled during mitochondrial fission with their tubular junction opened. This causes the release of proapoptotic factors, such as Cyt C and AIF. Our previous works also have shown that inhibition of mitochondrial fission can decrease the apoptosis on model of cerebral I/R injury in vivo and vitro[19,44,51]. In the current study, we revealed that propofol could inhibit mitochondrial fission during OGD and subsequent reperfusion injury in primary hippocampal cells. As a secondary messenger, Ca^2+^ not only plays an important role in physiological activities, but is one of the triggers involved in ischemic cell death, whatever the mechanism is[52]. Previous studies have shown that intracellular Ca^2+^ overload was closely related to I/R-induced apoptosis[53–54], and propofol could inhibit influx of extracellular Ca^2+^ by blocking voltage-gated calcium channels (VGCCs), similar to the effect of verapamil[55]. Moreover, it has confirmed that inhibiting of intracellular Ca^2+^ overload is an important mechanism for the protective effect of propofol on myocardium[56]. In the current study, we also determined that propofol could reduce the overload of Ca^2+^ in rat primary hippocampal neurons during I/R injury. In the model of cerebral I/R injury, propofol could inhibit Ca^2+^ overload by several mechanisms, including its antioxidant properties[57], which can inhibit the opening of calcium channel, and propofol can control the opening of receptor operated calcium channal (ROCC) by suppressing the combination of EAAs and NMDAR[52,58]. Additionally, propofol can block voltage-gated calcium channels (VGCCs) to inhibit intracellular Ca^2+^ overload as mentioned before.

In our experiment, we reasoned that the binding of Drp1-ser637 and Fis1 will be significantly reduced in the propofol treatment groups, which was supported by the results (Fig 4). We considered that intracellular calcium signal is strongly linked with mitochondrial fission because it can act on Drp1 to regulate the phosphorylation and dephosphorylation of specific amino acid sites, serine 616 and 637 based on previous studies [19,59–60].

Ca^2+^-dependent dephosphorylation of Drp1-Ser637 by CaN promotes mitochondrial fission and is involved in apoptosis, while dephosphorylation of Drp1-Ser616 promotes mitochondrial fission during mitosis[19]. CaN could be activated by the increasing intracellular Ca^2+^ concentration and then induce phosphorylation of Drp1-Ser637. Moreover, propofol (1μM-50μM) decreased the expression of Drp1-ser637 and Fis1, which also can reduce the combination of Drp1 and Fis1 directly during OGD/R.

There are a number of issues in this study that still are needed to be clarified. Firstly, although we have clarified that propofol could influence outer mitochondrial membrane division by regulating the activation of Drp1 in vitro cerebral I/R model, divisional mechanism of inner mitochondrial membrane is entirely different from that of outer mitochondrial membrane. Secondly, There are many other factors that can affect cytosolic calcium concentration, such as free radical production, NMDA receptor activation and excitatory amino acids generation, and we just explored the direct impact of propofol on cytosolic calcium overload in vitro cerebral I/R model. Finally, in the results of electron microscopy, lipid droplet were present in cytoplasm in propofol treatment group, but we didn't make a further study to clarify the effects of lipid droplets on neurons under laboratory conditions.

Despite these limitations, we have indicated that the neuroprotective mechanism of propofol in primary cultured hippocampal cells exposed to OGD/R in vitro might by inhibiting mitochondrial fission and suppressing the expression of mitochondrial proapoptotic factors Cyt C and AIF. Furthermore, we also clarified that propofol inhibited mitochondrial fission possibly by modulating the combination/expression of Drp1-ser367 and Fis1 in our experiment.

## Supporting Information

S1 File Western blot. (7Z)
